# Integrating endocannabinoid signaling, CCK interneurons, and hippocampal circuit dynamics in behaving animals

**DOI:** 10.1016/j.neuron.2025.03.016

**Published:** 2025-04-22

**Authors:** Shreya Malhotra, Florian Donneger, Jordan S. Farrell, Barna Dudok, Attila Losonczy, Ivan Soltesz

**Affiliations:** 1 Department of Neurosurgery, Stanford University, Stanford, CA, USA; 2 Department of Neurology, Harvard Medical School, Boston, MA, USA; 3 Rosamund Stone Zander Translational Neuroscience Center, Boston Children’s Hospital, Boston, MA, USA; 4 F.M. Kirby Neurobiology Center, Harvard Medical School, Boston, MA, USA; 5 Department of Neurology, Baylor College of Medicine, Houston, TX, USA; 6 Department of Neuroscience, Columbia University, New York, NY, USA; 7 Kavli Institute for Brain Sciences, Columbia University, New York, NY, USA; 8 Mortimer B. Zuckerman Mind Brain Behavior Institute, Columbia University, New York, NY, USA; 9 These authors contributed equally

## Abstract

The brain’s endocannabinoid signaling system modulates a diverse range of physiological phenomena and is also involved in various psychiatric and neurological disorders. The basic components of the molecular machinery underlying endocannabinoid-mediated synaptic signaling have been known for decades. However, limitations associated with the short-lived nature of endocannabinoid lipid signals had made it challenging to determine the spatiotemporal specificity and dynamics of endocannabinoid signaling *in vivo*. Here, we discuss how novel technologies have recently enabled unprecedented insights into endocannabinoid signaling taking place at specific synapses in behaving animals. In this review, we primarily focus on cannabinoid-sensitive inhibition in the hippocampus in relation to place cell properties to illustrate the potential of these novel methodologies. In addition, we highlight implications of these approaches and insights for the unraveling of cannabinoid regulation of synapses *in vivo* in other brain circuits in both health and disease.

## INTRODUCTION

The brain by dry weight is composed mostly of lipids, and endocannabinoids (eCBs) form a major class of lipid-derived information-carrying molecules. eCBs have important roles in a wide range of neuronal phenomena, including appetite regulation, temperature regulation, pain perception, brain development, learning and memory, and motor functions. In addition, the eCB system is implicated in a large number of psychiatric and neurological disorders, including epilepsy, pain, autism spectrum disorders, addiction, anxiety, psychosis, Alzheimer’s disease, Huntington’s disease, and Parkinson’s disease.^1–5^ The major neuronal receptor for eCBs is the cannabinoid type-1 (CB_1_) receptor. Reflecting the widespread involvement of the eCB signaling system in a diversity of cognitive, sensory-motor, affective, and homeostatic brain functions and dysfunctions, the CB_1_ receptor is one of the most abundant G-protein-coupled receptors (GPCRs) in the brain.^6^

The basic elements and key mechanistic features of the eCB system have been established for over two decades, mostly based on biochemical, immunocytochemical, *in vitro* electrophysiological, and behavioral studies.^1,6,7^ It has also been well recognized that while the expression of CB_1_ receptors is widespread across numerous cortical and subcortical areas, CB_1_ receptors at the microscopic scale are selectively localized on the presynaptic axon terminals of specific subpopulations of GABAergic and glutamatergic cells.^8–10^ The two major eCB ligands in the brain are the neuromodulatory lipids 2-arachidonoylglycerol (2-AG) and *N-*arachidonoyl-ethanolamide (anandamide, AEA).^11–13^ 2-AG is a full CB_1_ receptor agonist that is present at substantially higher concentrations (nmol g^−1^) than the partial agonist AEA (pmol g^−1^). Importantly, both eCBs are known to be highly labile molecules, due to the rapid action of specialized metabolic enzymes (see Box 1 for an introductory overview of the eCB signaling system).

In terms of synaptic actions of eCBs, *in vitro* electrophysiology studies carried out in culture systems and acute brain slices took advantage of the so-called depolarization-induced suppression of inhibition (DSI) and excitation (DSE) phenomena to reveal the cardinal properties of the eCB synaptic signaling system (Box 1).^23–25^ Namely, these research efforts showed that eCB signaling is a powerful, postsynaptic neuronal activity-dependent, retrograde process that results in a short-term depression of γ-aminobutyric acid (GABA) (in the case of DSI) or glutamate (DSE) release from nearby presynaptic axon terminals that express CB_1_ receptors. In DSI, a strong depolarization of the postsynaptic principal cell (typically to 0 mV for a second, delivered through the recording pipette by the experimenter) induces a transient (lasting several seconds) suppression of the incoming inhibitory synaptic events. Mechanistically, the strong depolarizing pulse activates voltage-gated calcium channels, leading to a prominent rise in intracellular calcium, which, in turn, activates postsynaptic membrane-bound enzymes that synthesize eCBs from their phospholipid precursors. The newly generated eCBs are then thought to be released from the activated postsynaptic neuron through a still-debated mechanism^34,35^ to retrogradely reach and activate the presynaptic CB_1_ receptors to decrease GABA release through the inhibition of calcium channels. Such *in vitro* studies also revealed that the basic features of DSE (e.g., its retrograde nature and CB_1_ antagonist sensitivity) are similar to those of DSI, although DSE in cortical circuits typically requires a longer postsynaptic depolarization (7–10 s) to induce it, at least with somatic single-cell recordings. Regardless of whether one considers DSI- or DSE-sensitive axon terminals, a key feature of the eCB signaling system is that CB_1_ receptors are heterogeneously expressed across cell types. For example, immunocytochemical studies, paired patch clamp recordings in acute slices, and pharmacological manipulations revealed that among GABAergic interneurons (INs), presynaptic CB_1_ receptor expression is highest in INs that co-express cholecystokinin (CCK). In particular, GABA release from perisomatically targeting basket cells (BCs) that express CB_1_ and CCK (CCKBCs) has been found to be exquisitely sensitive to eCBs in various brain regions, including the hippocampus, amygdala, and neocortex.^8,36,37^ Interestingly, eCBs and CB_1_ receptors are also involved in non-canonical signaling pathways. For example, 2-AG has been shown to potentiate GABA_A_ receptor activity at low GABA concentrations independent of CB_1_ receptors,^38,39^ while CB_1_ receptors are also modulated by non-classical ligands, such as pregnenolone.^40,41^

Despite the availability of detailed knowledge about the molecules and neuronal compartments involved in the eCB signaling system, how this molecular signaling pathway actually functions in particular synaptic-cellular circuits in behaving animals has remained incompletely understood, raising many fundamental questions. For example, can eCBs be generated in postsynaptic cells in response to physiological neuronal activity during normal behaviors (e.g., locomotion), or do they require unnaturally large stimuli similar to the depolarizing pulses used in DSI experiments in acute brain slices? Which eCB species is actually generated postsynaptically during the continuously fluctuating neuronal activity that typically accompanies natural behaviors? And are the eCBs generated in sufficient quantities by the active postsynaptic cells during physiologically relevant brain states to reach CB_1_ receptors on the presynaptic GABAergic terminals that impinge on them? Does a DSI-like suppression of inhibitory synaptic events exist *in vivo*? What are the neuronal dynamics of the CB_1_-expressing CCKBCs during naturally occurring brain states? And, finally, can CB_1_ receptor-dependent control of GABA release influence neuronal coding properties? In the current review, we primarily focus on the CA1 circuit of the hippocampus to illustrate how neurotechnological advances in the last few years have enabled researchers to answer some of these long-standing, fundamental questions about the eCB system in the neuronal circuits of behaving animals. These recent results highlight exciting new opportunities for research efforts aimed at developing an integrated, multi-scale understanding of molecular-level eCB signaling pathways engaged during natural behaviors at specific synapses under normal conditions and in psychiatric and neurological disorders.

## MOLECULAR TRACKING OF eCB FLUCTUATIONS IN THE HIPPOCAMPUS *IN VIVO*

### Development and validation of a novel eCB sensor

As mentioned above, biochemical and electrophysiological assays have suggested that a key property of the eCB system is the lability of its ligands, with signaling occurring over a timescale of seconds and over distances spanning just tens of micrometers.^23,42^ However, the majority of conventional, “gold standard” approaches used to study this system typically lack the spatiotemporal specificity that it requires. For example, post-mortem biochemical analyses of eCBs, typically measured with liquid chromatography and mass spectrometry, cannot capture potentially rapid fluctuations of eCBs during behavior. Even in the case of seizures, which are expected to capture supraphysiological levels of eCBs and are often used for validation of activity dependence, studies have yielded inconsistent results.^43–46^ *In vitro* electrophysiological experiments, such as DSI, have provided more clues on the spatiotemporal dynamics of eCBs but are indirect measures that, until recently, lacked *in vivo* validation (see below). Thus, a fundamental barrier to answering some of the key questions posed above has been the lack of a highly adaptive molecular tool that functions at timescales relevant for fluctuating neuronal activity *in vivo*.

**Figure 1.**
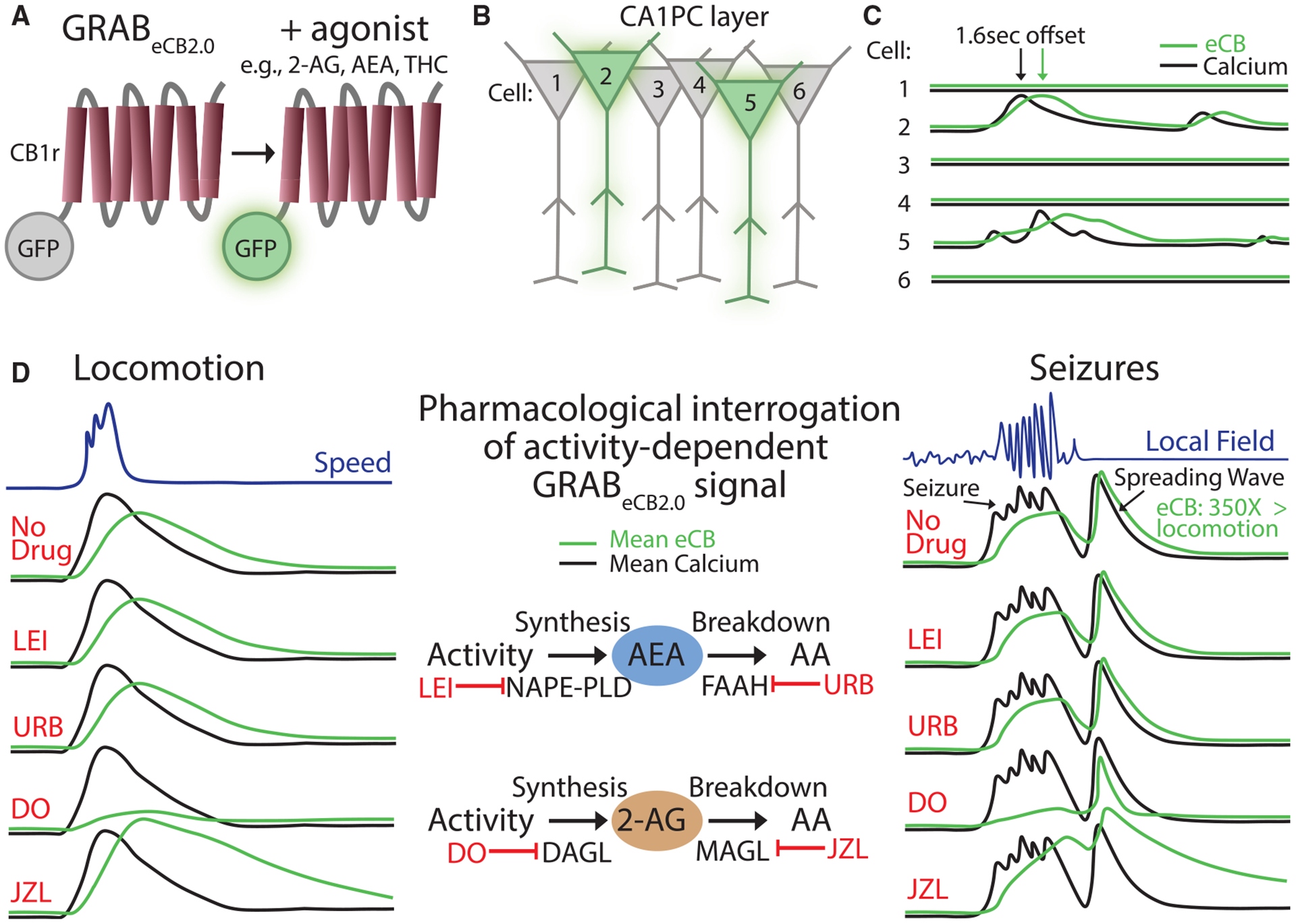
Characteristics of eCB dynamics *in vivo* (A) Cartoon depicting GRAB_eCB2.0_ as a GPCR conjugated to green fluorescent protein (GFP) with and without agonist. Upon ligand binding, the sensor increases GFP fluorescence. (B) Active neurons (cells 2 and 5) in the CA1 principal cell layer (CA1PC) are associated with an increased eCB signal, denoted as a green haze, that does not spill over onto neighboring neurons. See (C) for the associated calcium and eCB traces taken from regions of interest segmented around PC soma during two-photon imaging. (C) Calcium and eCB traces from segmented neurons in (B). eCB traces are tightly coupled to calcium activity, both spatially and temporally, but there is a slight lag in the eCB trace, consistent with activity-dependent production. (D) Mean calcium and eCB signals from the CA1PC layer during physiological (locomotion; left) and pathological (seizure; right) activity with pharmacological interrogation of the signal. Drugs to block the synthesis and degradation of AEA and 2-AG were used to determine if the eCB signal depended on either or both of these endogenous ligands. Under both physiological and pathological conditions, inhibiting 2-AG synthesis and degradation, but not AEA, was associated with the reduction and augmentation of the eCB signal, respectively, highlighting a key role for 2-AG in activity-dependent eCB dynamics. Notably, seizures were associated with a spreading wave that accompanied postictal flattening on the local field potential. This signal was ~350 times greater than physiological eCB signal changes.

The recent development of the GPCR activation-based eCB2.0 sensor (GRAB_eCB2.0_) has overcome this limitation by allowing the detection of endogenous eCBs with fast kinetics.^47^ GRAB_eCB2.0_ consists of the CB_1_ receptor as its scaffold coupled to green fluorescent protein, such that a CB_1_ agonist increases the amount of fluorescence emitted (Figure 1A). GRAB_eCB2.0_ has similar pharmacological properties to the native CB_1_ receptor, such as increased concentration-dependent activity with endogenous, phyto-, and synthetic agonists and reversal of fluorescence activity with antagonists and cannabidiol (CBD).^48^ Moreover, with the lack of an intracellular signaling domain, recruitment of downstream effector proteins has not been observed and should therefore not interfere with cellular physiology at reasonable expression levels (note that, as with any sensor, some degree of agonist buffering will occur, highlighting the importance of avoiding overexpression).^47^ Furthermore, GRAB_eCB2.0_ has onset (1.6 s) and offset (11.2 s) kinetics on second-long time scales,^47^ which are well-suited to capture the dynamics of DSI and DSE and highlight its utility for the *in vivo* study of neuronal activity. The sensor shows high specificity for eCBs when tested for a variety of neurotransmitter and neuromodulator ligands and a robust fluorescence response at physiological eCB concentrations (half-maximal effective concentration or EC_50_ for 2-AG and AEA is in the micromolar and submicromolar range, respectively). Any potential limitations that may arise for certain applications, for example, in kinetics, sensitivity, or specificity, are likely addressable by improvements introduced with newer versions of the tool (e.g., versions of the eCB sensor that can differentiate between 2-AG and AEA).

GRAB_eCB2.0_ captures eCB elevations during electrical stimulation of cultured neurons, which are blocked by AM251, a CB_1_ receptor antagonist/inverse agonist. Interestingly, blocking the synthesis of 2-AG, but not AEA, successfully blocks the GRAB_eCB2.0_ signal, suggesting that 2-AG primarily underlies this signal.^47^ Similar results were also obtained from slices of mouse striatum, including the observation that blocking 2-AG degradation slows the decay and that no signal change was observed in mutant mice lacking the 2-AG synthetic enzyme, DAGL.^49^ These results are consistent with prior *in vitro* work demonstrating a crucial role of 2-AG in short-term and long-term depression and motor control^50,51^ and establish GRAB_eCB2.0_ as a useful tool for resolving 2-AG dynamics, setting the stage for its use *in vivo*.

### Dynamics and spatiotemporal specificity of eCB signaling during physiological neuronal activity *in vivo*

With the substantial *in vitro* validation outlined above, this tool was ready to be applied *in vivo*, either with fiber photometry^47,52^ or two-photon imaging.^3,47^ The first question concerned the relative contribution of 2-AG versus AEA during physiological neural activity in the hippocampus. Utilization of two-photon imaging in awake, behaving mice revealed increases in GRAB_eCB2.0_ activity in CA1 PCs, closely following the elevated calcium signal during locomotion with an approximately second-long delay^3^ (Figures 1B and 1C). Much like the *in vitro* work above, pharmacology was then used to dissect the contribution of AEA or 2-AG to this signal (Figure 1D). Blockade of 2-AG synthesis led to suppression of the GRAB_eCB2.0_ signal in PCs, while blockade of 2-AG breakdown led to a robust increase with a prolonged decay. Notably, similar signal changes with enzymatic manipulation of 2-AG were observed with fiber photometry at ventral hippocampal to amygdala synapses.^52^ Enzyme inhibitors specific for AEA, on the other hand, resulted in no changes in GRAB_eCB2.0_ signal.^3^ Therefore, these findings strongly suggest that 2-AG is the primary eCB generated in response to hippocampal neural activity *in vivo*.

The second question concerned the spatial specificity of the eCB signal carried by 2-AG in the CA1 *in vivo*. Importantly, the coupling between the calcium and the GRAB_eCB2.0_ signals revealed spatially precise eCB signaling, as indicated by the observation that the eCB signal in a given neuron was more correlated to that cell’s calcium signal than that of other nearby cells. These results suggest a remarkable spatiotemporal specificity of eCB signaling, confined to when and where neural activity occurs. We will return to the topic of the spatiotemporal specificity of eCB dynamics when we discuss eCB signaling shaping perisomatic GABAergic inhibition as a function of hippocampal PC activity during spatial navigation (see below).

### Utilization of GRAB_eCB_ for studying eCB signaling during pathological neuronal activity

Given the abundant expression of the eCB system at synapses throughout the brain, it is unsurprising that it is thought to be involved in a variety of significant psychiatric and neurological brain disorders, including autism, addiction, pain, psychosis, eating disorders, alcohol use disorder, epilepsy, and cannabis use disorder.^1–5^ Increasing evidence, backed by genetic studies, has suggested that the eCB system undergoes changes during various pathological processes, but the function and contribution of these changes to disease pathophysiology often remain unclear.^53–58^ Application of GRAB_eCB2.0_ to study activity-dependent eCB dynamics offers a promising method for a direct readout of its engagement in models of neurological disease. For example, acute seizures were shown to lead to an over 100-fold increase in both the calcium and the GRAB_eCB2.0_ signals in the CA1.^3^ Much like in the case of physiological activity, pharmacology revealed a crucial role of 2-AG in seizure-related GRAB_eCB2.0_ signal (Figure 1D). This signal decayed back to baseline within a minute of seizure termination, which explains how prior studies using conventional biochemical methods that require removal of the brain were unable to capture this increase in 2-AG.^43–46^ Finally, the use of two-photon imaging to resolve the spatial patterns of seizure-related 2-AG increases revealed that the calcium and eCB activity of the entire CA1 PC network was engaged during the seizure, highlighting a loss of spatial specificity,^3^ and was followed by a traveling wave in both signals that was often larger than the seizure *per se*^59^ (Figure 1D). Thus, visualizing when and where eCBs are released in pathophysiology can lead to important insights, such as the potential role of spreading depolarization, which has now been consistently observed in a range of seizure models,^60^ in hijacking the molecular machinery that drives eCB synthesis.

## CELLULAR DYNAMICS OF THE CB_1_ RECEPTOR-EXPRESSING INs ACROSS BRAIN STATES IN THE HIPPOCAMPUS OF BEHAVING ANIMALS

### Genetic access to CB_1_ receptor-expressing hippocampal BCs

Despite being the most abundant GPCR in the brain, CB_1_ receptor expression is highly specific. In fact, although there are numerous IN types in the hippocampus, CB_1_ receptors are known to be predominantly present on the axons of CCK-expressing INs,^8^ whereas other prominent classes, including parvalbumin- (PV) and somatostatin-expressing, do not express CB_1_ or express it at much lower levels. However, due to the previous lack of CCK IN-specific molecular markers (e.g., CCK and CB_1_ receptors are expressed not only by CCK INs but also by PCs, albeit at a much lower level), genetic access to CCK INs has been limited (Box 2). Therefore, until recently, our knowledge of the CB_1_ receptor-expressing, perisomatically projecting CCKBCs in the hippocampal area CA1 *in vivo* has largely been based on a small number of recordings (from 7 cells in total) in anesthetized rats.^61,62^

One promising solution to this limitation was to combine single-cell resolution two-photon imaging of broad IN populations *in vivo* with *post hoc* immunocytochemical cell type identification.^70,71^ Acousto-optic deflection (AOD) microscopy-based three-dimensional (3D) *in vivo* calcium imaging of diverse GABAergic cells labeled in mice expressing Cre recombinase under the control of the vesicular GABA transporter (VGAT)^70,71^ provided key insights into CCK IN dynamics in awake, behaving animals (as discussed below). However, this approach did not distinguish CCK IN subtypes, including dendritically targeting CCK INs and CCKBCs with distinct eCB sensitivity and function (see below), and the two non-overlapping subtypes of CCKBCs that express either vasoactive intestinal polypeptide (VIP) or vesicular glutamate transporter type 3 (VGLUT3).^72,73^

A major step forward in studying specifically CCKBCs *in vivo* occurred with the development of a new mouse line that allows for specific, genetic targeting of this cell type.^66^ Using single-cell transcriptomics, the gamma synuclein (Sncg) gene was found to be selectively expressed in subsets of CCK cells^74–76^ and was used as a specific marker to target CCK INs in the Sncg-IRES2-Flp (Sncg-Flp) mouse line^66^ (Box 3). Importantly, electrophysiological recordings revealed that most neurons labeled by this technique exhibited an accommodating firing pattern^66^ (Box 3 image, panel A), and axons of the labeled neurons were restricted to the pyramidal layer, indicating that they constitute primarily CCKBCs (and not dendritically targeting CCK cells). In addition, Patch-sequencing experiments revealed that most CCKBCs labeled in the CA1 belonged to the VGLUT3 subtypes, with a non-overlapping minority expressing VIP. The development of the Sncg-Flp mouse line therefore allowed the first selective, population-level study of CCKBCs *in vivo*.

### Activity dynamics of CB_1_ receptor-expressing CCKBCs *in vivo*

**Figure 2.**
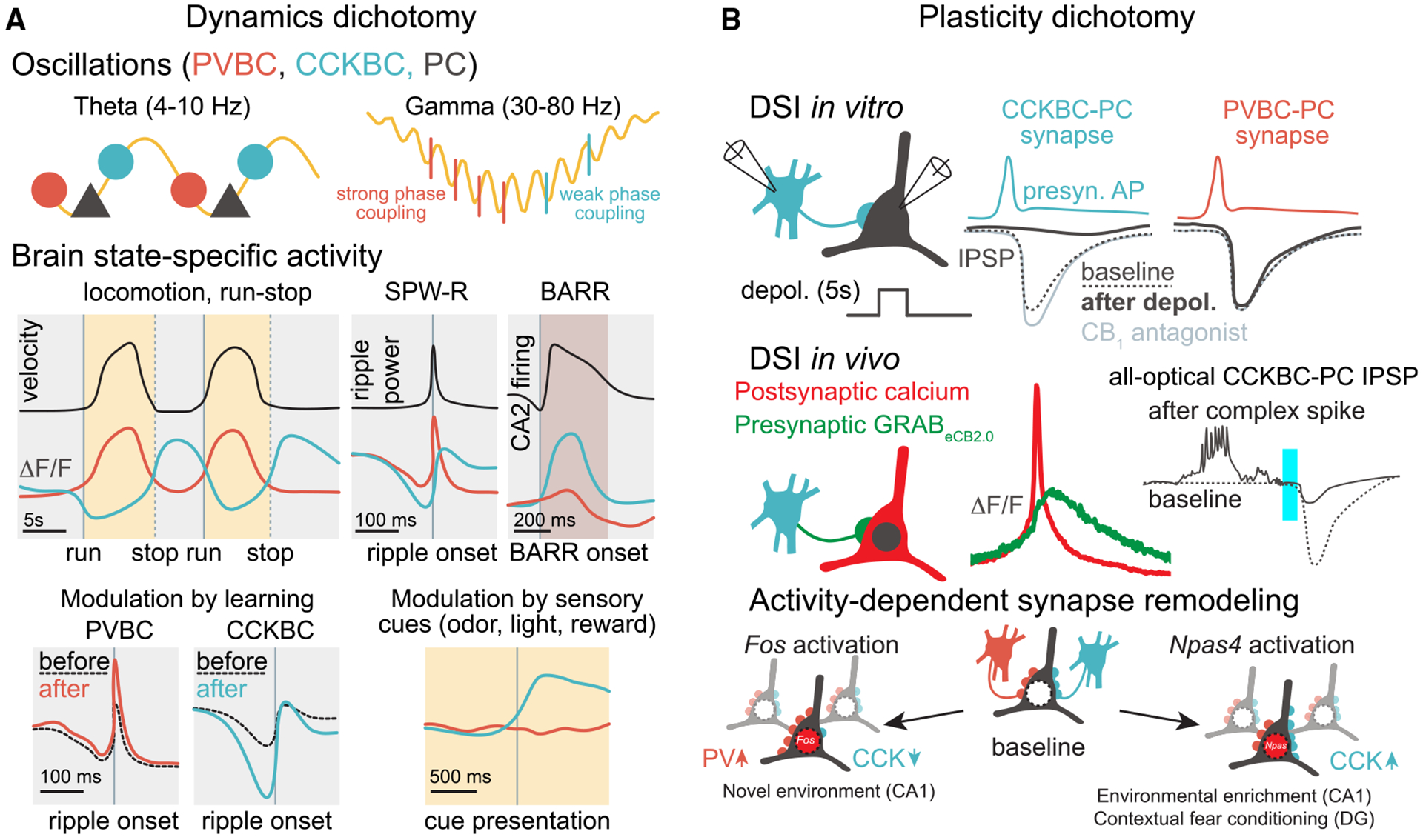
Basket cell dichotomy *in vivo* (A) Top: illustration of CCKBC and PVBC phase preferences during oscillations. Yellow lines symbolize field potentials. Markers indicate the relative timing of action potential firing (PVBC: orange; CCKBC: blue; PC: black). While PVBCs fire in the descending phase of the extracellular theta, CCKBCs fire in the ascending phase. During gamma oscillations, PVBCs fire in a strongly phase-locked manner after the trough of each gamma cycle, consistent with their role of driving gamma oscillations. By contrast, CCKBCs fire less consistently at variable phases. Bottom: illustration of cell type-specific activity dynamics. Curves in color symbolize average DF/F signals in an *in vivo* calcium imaging experiment. Vertical lines indicate the time of various events associated with brain state transitions. During locomotion (yellow shading), PVBCs are recruited while CCKBCs are suppressed. By contrast, after stopping (dashed vertical lines), CCKBCs are recruited. CCKBCs are suppressed before SPW-Rs, which emerge during immobility (gray shading) from a non-theta brain state, while PVBCs are recruited during the SPW-R. During sleep, barrages (BARR) of CA2 spiking recruit CCKBCs, while PVBCs are suppressed. When comparing SPW-Rs that are recorded before or after a goal-oriented learning session, PVBCs became more activated, while CCKBCs became more inhibited around SPW-Rs after learning. When sensory cues were presented during the task, CCKBCs were recruited compared with other INs, including PVBCs. (B) Top: illustration of an *in vitro* paired recording experiment to assess DSI. Note that in CCKBCs, but not in PVBCs, IPSPs in postsynaptic PCs are suppressed following PC depolarization (solid lines) compared with baseline (dashed lines). This suppression is mediated by eCBs, as indicated by its sensitivity to CB_1_ receptor antagonists. Middle: illustration of *in vivo* imaging experiments to assess DSI. GRAB_eCB2.0_ imaging shows eCB release following calcium transients in PCs (left). All-optical interrogation of IPSPs shows suppressed IPSP following PC depolarization (right). CCKBCs were optogenetically stimulated using blue light, while a genetically encoded voltage indicator was imaged to record changes in the membrane potential of postsynaptic PCs. Blue bar illustrates photoactivation of presynaptic CCKBCs. Optically evoked IPSPs were detected when the PC was quiet before the light pulse (dashed line), while IPSPs were suppressed if the light pulse followed a spontaneous plateau-driven complex spike in the PC (solid line). The depolarization associated with such events may release eCBs and induce DSI. IPSPs are not drawn to scale with action potentials. Bottom: immediate-early gene expression in PCs leads to BC type-specific inhibitory plasticity: while Fos activation leads to increased PVBC and decreased CCKBC synapse strength, Npas4 activation leads to increased inhibition by CCKBCs. Red nuclei symbolize activated cells (dark colors) compared with neighboring inactive cells (light colors). Round markers symbolize synapses.

Previous immunocytochemical and *in vitro* electrophysiological studies have suggested that CCKBCs may be tuning network excitability in response to behavioral states,^83^ but direct evidence in support of this inference was lacking. *In vivo* two-photon calcium imaging of the Sncg-Flp mouse line found that CCKBCs are indeed robustly modulated by behaviorally relevant brain state transitions.^66^ Interestingly, CCKBC activity was found to be largely anticorrelated on second-long time scales with surrounding network activity, including that of PV-expressing BCs (PVBCs) that exhibit complementary properties to CCKBCs in many synaptic and single-cell electrophysiological properties (Box 3 image, panel A).^77^ *In vivo* imaging studies using the Sncg-Flp line further highlighted this dichotomy. For example, during theta oscillations (4–10 Hz), which correlate with an engaged brain state and occur when an animal is running, the activity of CCKBCs generally decreases while PVBC activity increases.^66^ Extracellular silicone probe recordings from optotagged Sncg/CCKBC units indicated that there was not a complete suppression of CCKBCs during theta oscillations, in agreement with studies using *in vivo* juxtacellular recordings that have shown that the recruitment of CCKBCs and PVBCs takes place during distinct phases of the locomotion-associated theta rhythm,^61,84,85^ revealing a temporal segregation of PVBC and CCKBC activities even on the timescale of tens of milliseconds. At the end of the locomotion epochs (i.e., when the animal stops running and enters a period of immobility), theta oscillations give way to a period of irregular, low-amplitude local field potential (LFP) activity. During this locomotion-to-immobility transition time, there was a rapid decline in PC and PVBC activity, whereas CCKBC activity rose to a maximal level.^66^ The latter transient, maximal CCKBC activity dynamic is referred to as the “run-stop response”^70^ (Figure 2A). A few seconds after the animals stop running, sharp wave ripples (SPW-Rs), which are ensemble network events that are linked to memory replay and consolidation,^86^ start to appear on the LFP trace. During SPW-Rs, PVBCs were strongly recruited, whereas CCKBCs were suppressed. Taken together, these *in vivo* studies showed that the neuronal dynamics of PVBCs and CCKBCs are strictly complementary and alternating across different brain states and time scales. We refer to this complementarity as “inverse scaling” below (Figure 2A).

### Mechanisms of alternating sources of perisomatic inhibition *in vivo*

What may be the mechanistic underpinning of the inverse scaling between these two BC classes? As mentioned above, hippocampal PVBCs receive many excitatory inputs from both from local (CA1) and upstream (e.g., CA3) sources, which may explain why they can faithfully follow the overall activity levels within the PC populations. In comparison, the inversely correlated activity of CA1 CCKBCs with respect to the PVBCs predicts that this cell type may receive a unique pattern of excitatory and inhibitory inputs. Indeed, CCKBCs receive monosynaptic inhibition from PVBCs,^66^ which may explain the observed suppression during locomotion/theta oscillations and SPW-Rs during rest, when PVBC activity is high. In addition, CCK INs are innervated by local collaterals of theta-off/ripple-on (TORO) long-range projecting GABAergic cells, potentially contributing to the relative suppression of CCKBC activity during SPW-Rs.^82^

The excitatory inputs that activate CCKBCs remain poorly characterized. In general, CCKBCs receive weaker feedforward and feedback excitation and altogether fewer excitatory synapses compared with other INs, both in absolute numbers and relative to inhibitory synapses.^83,87^ The increase in CCKBC activity during the run-stop periods suggests that these cells play a crucial role in brain state transitions and potentially in currently incompletely understood spatial navigation-related circuit computations following the termination of locomotory bouts.^66^ What may drive CCKBC activity during the run-stop response? Is the run-stop response of CCKBCs purely due to the decrease in inhibition from PVBCs after the cessation of locomotion, or are there excitatory afferents to CCKBCs that may be driving it? Interestingly, the afferents within the CA1 that were particularly active at the time of the run-stop response were subsets of axons originating from the LEC and CA2^66^ (Box 3).

LEC inputs can include both glutamatergic and long-distance GABAergic afferents and are known to transmit information on reward-related and contextual cues to CA1 PC distal dendrites^88,89^ and also modulate INs that likely include dendrite-targeting CCK cells^90,91^ that may suppress dendritic spikes.^92^ However, whether CCKBCs are specifically innervated by LEC afferents and how such an innervation may shape CCKBC run-stop responses remains unclear. Although not yet directly shown, the CA2 may also provide inputs responsible for modulating CCKBC activity during the run-stop response. In CA2, a distinct population of PCs has been reported to represent current animal location during immobility and sleep, in association with a previously unidentified hippocampus-wide network pattern.^93^ Therefore, it is possible that CA1 CCKBCs are part of an immobility-associated network activated during the run-stop response. Moreover, during non-rapid eye movement (NREM) sleep, CA2 PCs initiate another previously undefined network event, firing a sustained barrage of action potentials (BARR).^94^ These non-theta, non-SPW-R events recruit CA1 CCKBCs, just like the run-stop response (Figure 2A). At the same time, other CA1 neurons, including PCs and PVBCs, are relatively silent. Interestingly, PCs that increased their activity the most during learning and subsequent SPW-Rs were the ones that were the most silenced during such barrages, possibly by CCKBCs. This relationship between past PC activity and present inhibition (i.e., the most active cells were more strongly inhibited) is distinct from DSI, which causes the most active cells to be the least inhibited, on the time scale of seconds. Whether the circuit mechanisms and cognitive functions of the run-stop response and CA2-driven sleep barrages are similar remains to be investigated in future experiments.

Most cortical regions, unlike CA1, are not targets of direct synaptic pathways from LEC or CA2, which raises the question of whether the inverse scaling of CCKBC and PVBC activity is specific to CA1. The answer is likely no, as cell types molecularly related to CCKBCs have inversely scaled activity patterns in the neocortex as well. In the primary visual cortex (V1), Sncg-expressing IN types, the homologs of CA1 CCKBCs, are inversely modulated during distinct brain states compared with PV INs.^95^ The brain state-specific activity modulation of V1 INs was strongly correlated to transcriptomic similarity. Moreover, the activity pattern of the so-called sleep down-state active (DSA) INs in the cortex^96^ also resembles that of CCKBCs in that DSA IN activity is inversely correlated to PCs and other INs (additional similarities include the observation that DSAs are transiently recruited when overall network activity drops and they receive relatively weak feedback excitation from the local circuit compared with other INs). While the Nkx2.1-positive DSAs in the neocortex are reported to be neurogliaform cells, not BCs,^96^ CCKBCs belong to the same broad transcriptomic group of cortical INs (Id2) and may be similarly modulated by brain state.^65^ Finally, in the hippocampal CA3 circuit, calbindin-positive SATB1 transcription factor-negative INs represent a major subset of immobility active INs,^71^ which have the same developmental origin as the CGE-derived CCK INs. Why would transcriptomic similarity predict similarity in brain state modulation?^95^ In addition to developmental specification, another potential explanation is that transcriptomically similar cell types share similar neuromodulator receptor repertoires and second messenger pathways. Considering the expression of various neuromodulatory receptors by CCKBCs, it is possible that the run-stop response is at least partially driven by cholinergic, noradrenergic, serotonergic, and/or dopaminergic inputs.^79,97^ Recent developments in a variety of GRAB_eCB_ sensors for these neuromodulators will help to better understand the interactions of neuromodulatory, GABAergic, and glutamatergic inputs shaping eCB and CCKBC dynamics.^98^

## HIPPOCAMPAL PLACE CELL PROPERTIES AND THE eCB SYSTEM

What may be the behavioral relevance of the eCB system in relation to CCKBC dynamics, and how can tools such as GRAB_eCB2.0_ and the unprecedented *in vivo* access to the CB_1_ receptor-bearing CCKBCs offered by the Sncg-Flp mouse line make it possible to study the latter question? As an animal navigates its environment, place cells in the CA1 fire at specific locations, known as place fields, and they collectively form a neuronal representation of space referred to as a “cognitive map.”^99^ Various studies have implicated the eCB system in spatial navigation. For instance, the use of synthetic cannabinoids has been shown to alter spatial memory and spike-timing coordination, although no alterations in location-specific place cell firing were detected.^100,101^ A major but previously not directly testable hypothesis has been that eCB-mediated inhibition of CCKBCs is likely involved in modulating place cell properties.^61^ This has specifically been hypothesized to occur through DSI, during which, as described above, *in vitro* observations revealed that strong depolarization of PCs ultimately leads to eCB-mediated suppression of GABA release for several seconds. Accordingly, increased place cell firing at place fields^99,102,103^ may lead to decreased GABAergic inhibition from CCKBCs through a DSI-like mechanism and a relative amplification of the excitability of only the currently active place cells, while other PCs would continue to receive normal levels of CCKBC-mediated GABAergic inhibition. In support of this proposed mechanism, *in vivo* studies indeed have found decreased inhibition corresponding to increased place cell activity^104–106^ but without direct evidence for the role of eCBs in this process.

Recently, two-photon imaging in behaving mice investigating the relationship between postsynaptic calcium signals in place cells linked to increased GRAB_eCB2.0_ fluorescence within the same regions of interest offered the long-elusive clues about the retrograde transfer of eCBs that could mediate DSI *in vivo*.^107^ Specifically, when animals were navigating a linear track lined with tactile cues, increased activity-dependent calcium signals in place cells at their place field locations were associated both with increased post- as well as presynaptic eCB signals at the same locations.^107^ These findings indicating the existence of place fields for eCB molecular signaling during spatial navigation strongly suggested the potential involvement of DSI in place cell coding but still fell short of showing any kind of modulation of inhibitory postsynaptic potentials (IPSPs) as a function of postsynaptic neuronal activity *in vivo*. Although the direct measurements of subthreshold, fast inhibitory activity in behaving animals seemed out of reach even as recently as a few years ago, advances in all-optical interrogation of circuit and cellular-synaptic properties using voltage imaging in combination with optogenetics^108,109^ now make it feasible to detect small hyperpolarizing voltage changes (i.e., IPSPs) in CA1 PCs not just in general but also in response to activation of specific subtypes of INs *in vivo* (Figure 2B). Previous studies using whole-cell recording *in vivo* have found that a particular PC firing pattern, plateau-driven complex spiking, plays an important role in synaptic plasticity and place cell formation,^103,110^ findings that recently have also been demonstrated using all-optical physiology.^108,109^ Importantly, plateau-driven complex spikes are large events associated with postsynaptic calcium entry and may therefore also lead to the synthesis and release of eCBs, which is consistent with the increased eCB signal at place fields. Indeed, the combination of voltage imaging and genetic access to CCKBCs provided by the Sncg-Flp mouse line demonstrated that CCKBC-evoked IPSPs in CA1 PCs were significantly smaller when preceded by plateau-driven complex spikes in the same PCs, in agreement with what would be expected from a DSI-like phenomenon^107^ (Figure 2B). Notably, mice lacking CB_1_ receptors in INs, which mostly affects CCK cells, displayed a widening of place fields. Furthermore, population-level encoding of the animal’s position was less accurate, indicating impaired place cell properties in the absence of eCB control of CCKBC synapses. These findings indicate that a DSI-driven phenomenon *in vivo*, relying on presynaptic CB_1_ receptors on GABAergic terminals, is crucial in regulating the precision of place cell firing and supporting spatial navigation.^107^

## ADDITIONAL COGNITIVE FUNCTIONS OF CCKBCs AND THE eCB SYSTEM

As described above, CCKBCs and the eCB system play important roles in cognitive functions. For instance, the increased activity of CCKBCs during the run-stop response suggests their potential involvement in implementing brain state transitions via changes in local circuit dynamics, and *in vivo* evidence of DSI suggests a role of eCBs in behaviorally relevant neural activity related to spatial navigation. Studies in the CA3 of the hippocampus suggest that CCK cells may also be involved in other cognitive functions, such as memory consolidation and selective attention. Indeed, using AOD 3D calcium imaging, CCK cell dynamics suggested that they may play a significant role in modulating SPW-Rs in the CA3.^71^ Specifically, the magnitude of inhibition of CCK cells *before* the SPW-R was correlated with the duration of the SPW-R. Interestingly, it was the magnitude of PVBC response *after* the SPW-R that was associated with the duration of the SPW-R. This study also demonstrated that spatial learning induces changes in CA3 inhibitory network dynamics such that PVBCs and CCKBCs become more activated and inhibited, respectively, around SPW-Rs after learning,^71^ further showcasing the dichotomy of CCK and PV cell dynamics *in vivo* (Figure 2A). Given that the duration of SPW-Rs is likely to be related to memory performance,^111^ these results further highlight the potential roles of CCK INs in cognitive processes.

Importantly, the Vancura et al.^71^ study also reported that CCK cells were preferentially activated in response to sensory cues, such as reward, light, and odor. Although we do not yet have direct evidence for the role of these cue-responsive CCK cells, they may significantly contribute to selective attention. Schaffer collaterals, originating from CA3 PCs and terminating on CA1 PC dendrites in the radiatum, are thought to be important for encoding and consolidating memories by driving SPW-Rs.^112–115^ Interestingly, a recent study found that Schaffer collateral axons in the CA1 were activated by specific sensory cues, but their activation was excluded from SPW-Rs if the associated cues were not spatially relevant.^116^ SPW-Rs therefore are preferentially biased to behaviorally relevant information and actively suppress irrelevant stimuli. In other words, as an animal navigates its environment and encounters a barrage of sensory information, only the most relevant cues, such as those pertaining to the location of food or important landmarks, are encoded during SPW-Rs.^116^ Moreover, in a different study using a virtual reality maze, CA1 CCK INs showed a strong modulation of activity depending not only on reward but also on the animal’s interaction with the maze (i.e., cells were activated when the virtual reality was turned off, and therefore sensory information was not salient to the task).^117^ Given that non-salient cues were actively inhibited during SPW-Rs and that CCK cells display the strongest cue responses in the CA3 during active exploration^71^ (Figure 2A), it is possible that these cells are modulating and filtering the CA3-CA1 circuit based on cue salience,^118^ potentially further extending the cognitive impact of CCK INs.

The plasticity of CCKBC synapses may also play a role in contextual memory. The consolidation of contextual fear memory involves synaptic plasticity to facilitate the reactivation of unique neuronal ensembles representing specific memories—these ensembles are frequently called memory engrams.^119^ The role of inhibitory synapse remodeling in these processes remains the focus of inquiry. Distinct activity patterns of PCs result in the activation of distinct immediate-early gene networks (Box 3 image, panel B), characterized by the expression of either Fos or Npas4.^120^ Interestingly, Npas4 activation enhances inhibitory inputs from CCK INs in the dentate gyrus and CA1,^120–122^ while Fos activation leads to enhanced PV IN inputs and reduced CCK IN synapses through a mechanism involving neuropeptides encoded by the Scg2 gene.^123^ Such inhibitory plasticity, and the activity of CCK INs, is required for the emergence of selective engrams and for discrimination during recall.^120,124^ As the Fos and Npas4 regulators can be activated in different cells at the same time, these mechanisms likely contribute to the development of circuits preferentially innervated by either CCK or PV INs.

## OUTSTANDING QUESTIONS

**Figure 3.**
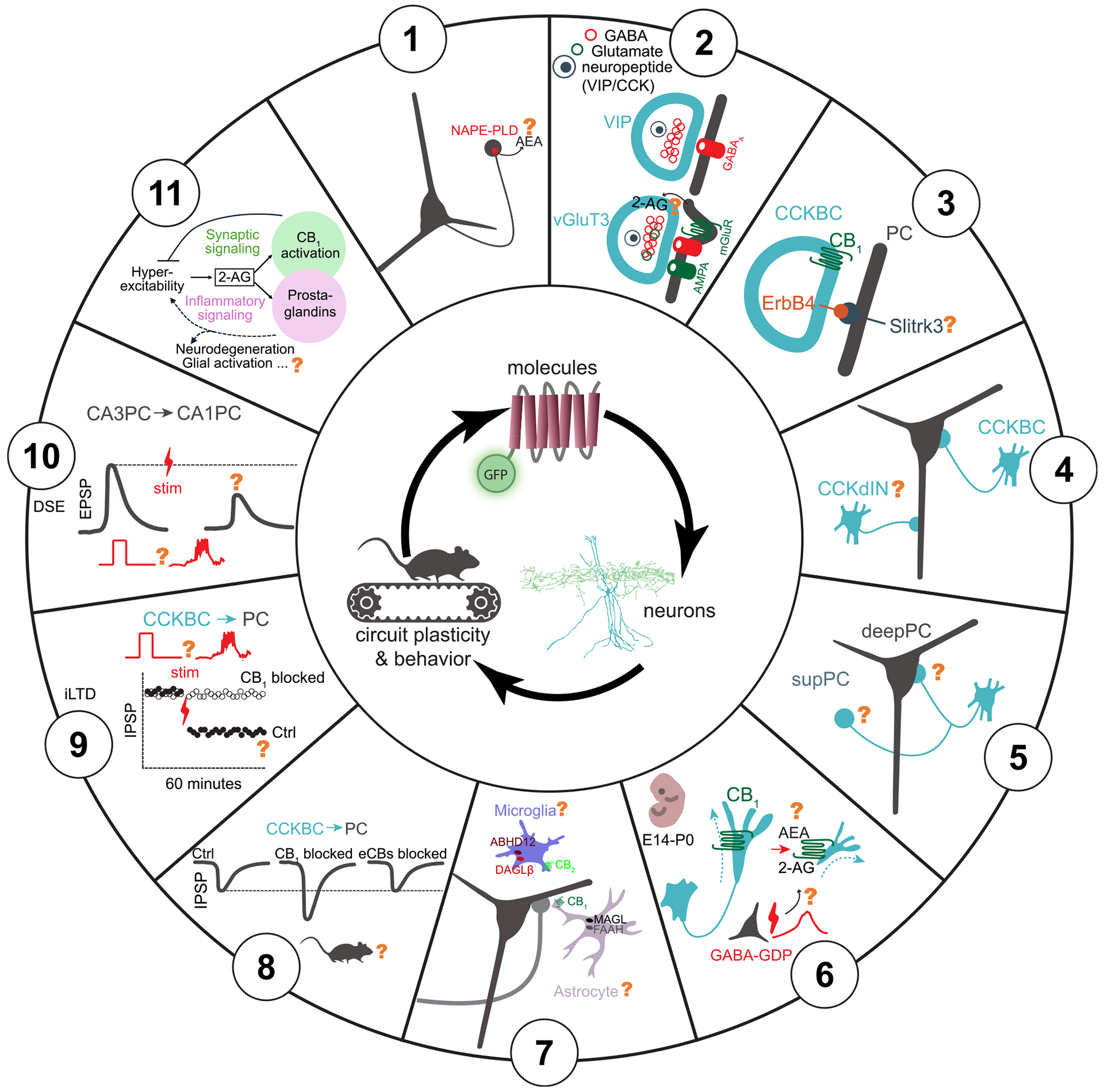
Outstanding questions regarding multi-scale integration of eCB signaling in behaving animals The panel numbers correspond to the outstanding questions section within the main text. The illustrated drawings are schematic depictions of key topics discussed and referenced in the main text. 1. When and where is AEA synthesized, where does it act, and how long does its activity persist *in vivo*? Schematic shows a representative image of a neuron expressing NAPE-PLD in its axon terminals. 2. Does the co-release of GABA with glutamate and/or neuropeptides (CCK or VIP or both) occur *in vivo* following physiological level of activity? Does glutamate co-release trigger retrograde eCB signaling in VGLUT3 CCKBCs? 3. What are the postsynaptic molecules that interact with the receptor tyrosine kinase ErbB4 to determine the specific targets of CCKBC synapses? Slitrk3 is a potential candidate. 4. What are the *in vivo* roles of CB_1_ receptor- and CCK-expressing, dendritically projecting INs (CCKdIN) in regulating dendritic information processing? 5. Do CCKBCs preferentially regulate distinct subclasses of PCs such as the superficial versus deep CA1 PCs in behaving animals? 6. How does eCB signaling regulate axonal pathfinding and shape spontaneous GABAergic network dynamics (GABAergic GDPs) in developing neuronal circuits *in vivo*? 7. What are the physiologic and pathologic roles of astrocytes and microglia in the eCB system? Astrocytes contain CB_1_ receptors, as well as eCB degradation enzymes MAGL and FAAH. Meanwhile, microglia likely contain CB_2_ receptors and eCB the synthesis and degradation enzymes DAGLβ and ABHD12. What are the roles of these receptors and machinery in shaping eCB signaling during behavior? 8. Does CB_1_-mediated tonic inhibition of GABA release exist *in vivo*, and does it influence the probability of GABA release in a functionally relevant manner? The schematic illustration depicts how blockade of the intrinsic, ligand-free activity of the CB_1_ receptor with an inverse agonist leads to a robust increase in the unitary evoked IPSCs originating from a presynaptic CCKBC and recorded from a postsynaptic PC. By contrast, blocking eCB ligand synthesis does not have similar effect. 9. The schematic depicts iLTD that is absent when CB_1_ receptors are blocked. What are the physiological conditions (e.g., theta-burst firing) that can induce long-term depression of cannabinoid-sensitive inhibition, and what are its functional effects *in vivo*? 10. Can physiologically occurring dendritic plateau potentials trigger DSE in behaving animals, and does it play a role in BTSP? 11. How does neuronal hyperactivity, and the resulting increase in 2-AG, interact with synaptic signaling (through CB_1_ activation) and inflammatory signaling (through prostaglandin synthesis) in disease processes?

Finally, we briefly summarize what we believe are some of the most important questions (Figure 3) concerning the eCB system that the field can now begin to address by taking advantage of some of the new technical advances discussed above. We begin with questions related to molecules involved in eCB signaling, move to those pertaining to cellular circuits, and finally end with questions with the most behavioral and pathological relevance.

### 1. Dynamics of AEA

Previous measurements obtained *in vivo* from several groups utilizing GRAB_eCB2.0_ have found a key role for 2-AG in the underlying phasic signal changes.^3,47,49^ It is still unclear how AEA dynamically fluctuates, both spatially and temporally, as it relates to ongoing neural activity. Since AEA levels are generally orders of magnitude lower than 2-AG, overcoming this challenge may come from the development of new sensors designed for higher AEA sensitivity (GRAB_eCB2.0_ EC50 for AEA is between 200 and 800 nM). Another consideration is that AEA may have fundamentally distinct spatiotemporal dynamics. One proposed model for AEA function, which is a partial CB_1_ receptor agonist, is that it acts as a slow, tonic signal that sets the tone of the CB_1_ receptor, whereas the full agonist, 2-AG, fine-tunes CB_1_ receptor activity with high temporal precision.^125–127^ At perisomatic CCK synapses, this interaction may be more complex, since FAAH inhibitors that elevate AEA levels can regulate phasic 2-AG synthesis *via* transient receptor potential vanilloid type 1 (TRPV1) receptors, while CB_1_ receptors on dendritically targeting CCK INs are not regulated by FAAH inhibition.^128^ Thus, the temporal dynamics and synapse-specific functions of AEA are unclear. In addition to the potentially unique temporal dynamics of AEA, it is also possible that circuits that have a greater bias for AEA production have not been investigated yet. Within the hippocampus, for example, only the CA1 has been studied, but levels of the biosynthetic enzyme for AEA, NAPE-PLD, are higher in the CA3 and DG.^129^ At a subcellular level, it is important to note that NAPE-PLD is expressed in dentate granule cell axon terminals, which do not have CB_1_ receptors.^130^ Thus, AEA may not function as a traditional retrograde transmitter (i.e., from soma/dendrite to axon), and, as highlighted above, likely has other molecular targets than the CB_1_ receptor, such as the TRPV1 receptor. Thus, unlike 2-AG, which has significant *in vitro* data to formulate hypotheses for how it might act *in vivo*, a hypothesis for when and where AEA is synthesized, where it acts, and how long its activity persists is less straightforward.

### 2. Co-release of neuroactive substances from CCKBCs

A defining feature of CCK INs is the expression of the CCK neuropeptide, which is present in dense-core vesicles in these cells. *In vitro*, exogenously applied CCK has profound effects on inhibition, including a robust activation of PVBCs and the CCK receptor-mediated synthesis and subsequent release of eCBs.^131,132^ The eCBs depress GABA release from CCKBC terminals, amplifying perisomatic inhibition originating from PVBCs at the expense of CCKBCs.^131,132^ Whether CCK can be co-released with GABA from CCK INs during behavior remains to be established, likely aided by the recent availability of the GRAB_eCB_ sensor.^133^ In addition, as briefly mentioned above, a subset of CCK INs (including both CCKBCs and dendritically targeting CCK INs) co-express VGLUT3 (encoded by the Slc17a8 gene), and this subset appears to be mutually exclusive with VIP-expressing CCK INs based on immunohistochemistry.^72,73^ Further evidence indicates that VGLUT3-expressing CCKBCs constitute a distinct cell type where glutamate-GABA co-release can occur at their output synapses with potentially unique functional roles.^73^ Interestingly, the Sncg-Flp line mostly labels VGLUT3 CCKBCs (see above), and the role of ErbB4 in CCKBC wiring (see below) also appears to be specific to VGLUT3-expressing cells, as ErbB4 expression is only marginally present in VIP-expressing CCKBCs.^134^ In the amygdala, VGLUT3-containing CCKBCs form specialized, invaginated synaptic structures on postsynaptic PCs, rich in metabotropic glutamate and CCK receptors, and molecules involved in the downstream eCB-synthesizing enzymatic pathway.^135^ Thus, co-release of glutamate may trigger retrograde eCB signaling, forming a negative feedback loop to suppress GABA release after particularly high presynaptic activity at CCKBC synapses. Furthermore, in the hippocampus, co-released glutamate can also activate postsynaptic AMPA receptors at VGLUT3 CCKBC synapses, which may render these synapses paradoxically excitatory under pathological conditions such as in epilepsy.^73^ However, the *in vivo* existence and roles of CCK and glutamate co-release from CCKBCs remain an open question.

### 3. Role of ErbB4 in the integration and function of CCKBCs in hippocampal networks

ErbB4, a receptor tyrosine kinase, has been shown to be essential for the successful integration of CCKBCs and PVBCs into hippocampal networks,^136^ including the establishment and refinement of their excitatory inputs.^6,134,137–141^ Specifically, ErbB4 expression in CCKBCs is crucial for their synaptic connections with both PCs and PVBCs.^134^ A key question concerns the identity of the postsynaptic adhesion molecules that interact with ErbB4 to determine the specific targets of CCKBC synapses. One potential candidate is the transmembrane protein Slitrk3, recently shown to interact with ErbB4 and promote the formation of inhibitory synapses on PCs.^142^ Importantly, specific deletion of ErbB4 in VGLUT3 CCKBCs during development has been shown to alter inhibitory signaling onto PCs, reduce the power of theta oscillations during exploratory behavior, disrupt spatial coding by place cells, and selectively impair spatial learning and memory in adult mice.^134^ Many of these disruptions likely occur due to impaired eCB signaling. These findings are particularly intriguing, especially the role of VGLUT3 CCKBCs in modulating theta oscillations, a role previously mainly attributed to PVBCs. Given that, as discussed above, VGLUT3 CCKBCs can release both GABA and glutamate onto postsynaptic PCs,^72,73,135,143,144^ it is plausible that it is this co-release mechanism that endows CCKBCs with unique computational properties that could be crucial for modulating theta oscillations.

### 4. Functions of dendritically projecting CB_1_ receptor-expressing INs

Different compartments of PCs receive unique compositions of local and long-range inputs. Recent studies have shown that different dendritic compartments exhibit distinct activity and plasticity dynamics during place field formation.^109,145,146^ Although most attention has been focused on CCKBCs whose perisomatic synapses are implicated in shaping place cell properties during spatial navigation through a DSI-like mechanism (as discussed above), dendritically targeting CCK INs likely also impact information processing and may be regulated by the eCB system in distinct ways. Dendritically targeting CCK INs are composed of at least seven identified subtypes, distributed throughout all layers of CA1.^147,148^ There is much left to be discovered about most of these subtypes, as evidenced by the very small sample sizes (usually only 1–2) for *in vivo* recorded and identified cells for each of these dendritically projecting CCK IN subtypes. It is likely that dendritically targeting CCK INs have eCB signaling properties and *in vivo* activity dynamics that are distinct from CCKBCs. For example, CA1 Schaffer collateral-associated CCK INs that project to the stratum radiatum in the CA1 have been shown to display no or considerably weaker DSI, CB_1_ receptor-dependent tonic inhibition of GABA release, and metabotropic glutamate receptor activation-induced, CB_1_ receptor-mediated depression of GABA release. This is the case in spite of the prominent presence of all members of the eCB-synthesizing molecular machinery in the dendrites of postsynaptic principal cells and the expression of functional CB_1_ receptors in the axon terminals of these INs.^10^ Furthermore, molecularly identified CCK INs, which include both CCKBCs and dendritically targeting INs, exhibit a more heterogeneous velocity modulation profile compared with immobility active CCKBCs, suggesting that at least some dendritically projecting CCK INs are active during locomotion.^70^ Therefore, dendritically projecting CCK INs may have differential effects on behavioral readouts and eCB-mediated processes. Indeed, these cells are well-positioned to modulate PC activity in an input-specific manner, since different compartments of PCs receive distinct excitatory inputs from distinct intra- and extrahippocampal areas.^147^ Along these lines, dendritically projecting CCK INs have been suggested to be involved in gating dendritic spikes driven by LEC inputs.^92^

### 5. eCB modulation of perisomatic inhibition onto heterogeneous CA1 PC populations

As previously discussed, CCKBCs and PVBCs modulate CA1 PCs in opposite yet complementary ways.^66^ This dichotomy is exacerbated by their differential synaptic connectivity with respect to the heterogeneity present in the CA1 PC population itself, characterized by differences in PC soma positioning along the radial axis (from superficial to deep layers), distinct neurochemical markers, and long-range projection patterns.^149–151^ Surprisingly, it has been discovered that PVBCs exert approximately 3-fold stronger inhibition onto CA1 PCs located in the deep compared with superficial layer of the stratum pyramidale.^152,153^ Additionally, superficial PCs more frequently provide excitatory inputs to PVBCs than their deep counterparts. PVBC-to-PC inhibition also segregates along PC projection patterns. For example, PVBCs preferentially innervate PCs projecting to the amygdala compared with their prefrontal cortex-projecting neighbors but receive preferential excitation from PCs projecting to the prefrontal cortex and much less from the amygdala-projecting PCs.^152^ Whether such preferential innervation of PCs also applies to CCKBCs is not yet established, with one study finding no overt selectivity,^152^ whereas another report suggests that CCKBCs may primarily inhibit superficial CA1 PCs.^153^ Interestingly, CCKBCs do indeed show robust selectivity for postsynaptic principal cell populations in layer II of the medial entorhinal cortex.^154^ These observations raise multiple questions, for example, related to the additional computational properties potentially provided by eCB signaling in a selective manner to certain PC populations preferentially targeted by CCKBCs in neuronal circuits. Exploring such questions could provide valuable insights into the distinct roles of CCKBCs and PVBCs and may also yield novel molecular-genetic interventional tools to study their impact on learning and memory.

### 6. Neuronal activity-dependent roles of the eCB signaling system in the developing brain *in vivo*

The eCB signaling system plays a variety of crucial roles in the developing nervous system, from lineage segregation of stem cells and excitatory and inhibitory synapse positioning to the refinement of synaptic functions and the control of adult neurogenesis.^155–157^ Indeed, the highest levels of CB_1_ receptor expression occur as synaptic connectivity is established during embryonic (PCs) and early postnatal (GABAergic INs) development.^155^ Pharmacological interference with the CB_1_ receptor suggests that these receptors modulate synchronous GABAergic depolarizing network events, known as giant depolarizing potentials (GDPs), in the developing hippocampus.^158^ The emerging novel tools and approaches described in this review may also shed light on how the eCB-mediated activity-dependent feedback mechanisms shape spontaneous coincident neuronal population dynamics, including those linked to externally generated sensorimotor activity during early postnatal development.^159^

### 7. eCB signaling in glia

Although we have focused on the eCB system in neuronal pathways, growing evidence suggests that astrocytes and microglia play important roles related to eCB activity, function, and metabolism.^160,161^ Astrocytes, the most abundant cell type in the central nervous system, are classically known for their role in neuronal support, homeostasis, and synapse function.^162,163^ These cells express functional CB_1_ receptors, which lead to astroglia calcium increases that can subsequently spread along the cell and lead to the release of neuroactive substances referred to as “gliotransmission” (typically involving ATP and glutamate), as well as short- and long-term potentiation.^164–169^ Additionally, astrocytes express TRP channels and other GPCRs that may be modulated by cannabinoids.^170–172^ In terms of metabolism, astrocytes notably express MAGL and FAAH, enzymes that are responsible for 2-AG and AEA breakdown, respectively (Box 1), and are likely important for attenuating neuronal cannabinoid signaling at the so-called “tripartite” synapse involving pre- and postsynaptic structures and astrocytic processes.^173–177^ eCB signaling in astrocytes likely also plays a significant role in inflammation, based on studies suggesting that exogenously applied AEA and synthetic cannabinoid analogs can have anti-inflammatory effects.^178–182^ Along the same lines, inhibitors of MAGL have been shown to reduce lipopolysaccharide-induced inflammation.^175,183^ However, the exact pathways for these findings, as well as their implications *in vivo*, remain to be discovered. Furthermore, although there is some evidence that astrocytes may release 2-AG and AEA, it remains an open question whether these eCBs have functional relevance.^184–187^

Microglia, known for their immune functions in the brain, likely also contribute to eCB signaling and modulation. Unlike in astrocytes, however, the functionally relevant expression of CB_1_ receptors in these cells is not fully established.^161,188,189^ Interestingly, microglia do express *Cnr2* transcripts, coding for CB_2_ receptors, at higher levels than neurons.^190^ Studies suggest that microglial CB_2_ receptors may facilitate communication between neurons and microglia and modulate glutamatergic neurotransmission.^191–197^ Furthermore, cannabinoid signaling, especially CB_2_ receptor expression, in microglia is activity dependent and likely contributes to modulation of neuroinflammation.^198^ Findings suggest reduced levels of infiltrating macrophages as well as a reduced proinflammatory drive upon CB_2_ receptor deletion.^199^ Modulation of eCB-mediated neuroinflammation likely also occurs through microglia expression of eCB synthesis and degradation enzymes, diacylglycerol lipase-β (DAGLβ) and α/β-hydrolase domain-containing 12 (ABHD12).^200^ Future studies can further explore these findings *in vivo* to dissect eCB-mediated microglial involvement in neuroinflammation and the modulation of neurotransmission.

### 8. Tonic inhibition of GABA release by CB_1_ receptors *in vivo*

In addition to their role in DSI-related activity-dependent short-term plasticity, CB_1_ receptors are known to also modulate GABA release from CCKBCs in a time-invariant manner.^128,201^ This occurs through their intrinsic, constitutive, ligand-free activity, most likely related to the ability of GPCRs to flip into an active conformation with some non-zero probability even in the absence of the ligand.^128,201^ This tonic regulation of GABA release by CB_1_ receptors (see also panel G in the Box 1 image) has been shown in *in vitro* studies to be a powerful regulator of the probability of release at inhibitory synapses of CCKBCs that can even lead to “silent GABAergic synapses.”^10,28–30^ At such “silent” GABAergic synapses, the probability of release is typically close to zero (i.e., a presynaptic action potential seems to evoke no postsynaptic responses in paired recordings *in vitro*), but it can be dramatically increased in the presence of an inverse agonist that blocks the constitutive GPCR activity.^128,201^ One promising indication that tonic eCB signaling alters neuronal activity during behavior comes from comparing neuronal activity between two visual regions with quantitatively different tonic eCB signaling.^202^ In the secondary visual cortex (V2), a relatively strong cannabinoid tone (observed *in vitro*) seems to coexist with relatively high spontaneous PC activity *in vivo*. By contrast, in primary visual cortex (V1), a lower cannabinoid tone and consequently stronger inhibition (assessed *in vitro*) seem to accompany lower spontaneous PC activity *in vivo*. Furthermore, treatment with the CB_1_ receptor antagonist/inverse agonist AM251 eliminates such differences *in vivo*,^202^ indicating that the cannabinoid tone may indeed modulate PC firing rates. Importantly, however, direct evidence for the existence of tonic inhibitory control of GABA release by CB_1_ receptors *in vivo* is still lacking, and it is also not known as to what degree it can depress GABA release in behaving animals. A related question is whether the selective presence of the tonic control of GABA release by CB_1_ receptors at perisomatically but not dendritically targeting CCK INs (see above) observed *in vitro*^10,128,201,203^ also applies to the *in vivo* situation. The importance of a better understanding of the *in vivo* status of the CB_1_ receptor-dependent tonic control of GABA release is underlined by the fact that it was found to be selectively disrupted in animal models of autism in acute hippocampal slices, without corresponding changes in DSI.^204^ These questions related to the CB_1_ receptor tonic activity *in vivo* can be explored in knock-out mouse models that lack the synthesizing enzyme for 2-AG (diacylglycerol lipase-α [DAGLα]−/−)^16,17^ or AEA (NAPE-PLD−/−).^205^

### 9. eCB-mediated longer-term plasticity of inhibition

*In vitro* experiments revealed that, on a longer time scale than DSI (on the order of minutes), cannabinoid-sensitive inhibition can also be tuned by long-term depression (referred to as iLTD). In CA1 PCs, theta-burst firing, designed to mimic *in vivo* patterns of place cell activity, triggered retrograde eCB signaling that persistently suppressed presynaptic inhibition.^206^ Interestingly, by contrast to DSI, which is expressed by a G-protein-mediated suppression of voltage-gated calcium channels and vesicle release, iLTD also requires presynaptic protein synthesis downstream to CB_1_ receptor activation.^207,208^ Whether iLTD-like long-term plasticity mechanisms contribute to place cell formation and maintenance during spatial navigation in the hippocampus *in vivo* remains to be investigated. Interestingly, a unique form of CA2 iLTD has been implicated in social memory formation *in vivo*.^209^

### 10. DSE during natural behaviors

While DSE has been demonstrated under *in vitro* conditions in a variety of brain circuits,^25,210–214^ it remains to be investigated if DSE exists in the intact brain *in vivo* during natural behaviors. A related and intriguing question is whether and how eCB signaling plays a role in behavioral timescale plasticity (BTSP). Intuitively, the large amplitude calcium channel- and NMDA receptor-mediated dendritic plateau potentials that induce BTSP should lead to eCB release and DSE at glutamatergic synapses. Therefore, DSE may preferentially suppress synapses that are active *after* dendritic plateaus and, in turn, regulate the shape of the bidirectional plasticity kernel of BTSP.^215^

### 11. Pathological eCB signaling in brain disorders

In agreement with the wide distribution and high density of CB_1_ receptors in a variety of reward, habit, and cognition-related circuits, eCB signaling has been implicated in a large number of neurological and psychiatric disorders, including epilepsy, pain, autism spectrum disorders, addiction, cannabis use disorder, eating disorders, anxiety, psychosis, aging, Alzheimer’s disease, schizophrenia, Huntington’s disease, Parkinson’s disease, and many others.^1–5^ Accordingly, key targets in the eCB system may provide therapeutic opportunities for such disorders. For instance, inhibition of 2-AG hydrolysis may be beneficial for certain epilepsies and neuroinflammatory diseases. 2-AG is primarily hydrolyzed by MAGL in presynaptic neurons and a smaller percentage by α/β-hydrolase domain-containing 6 (ABHD6) in postsynaptic neurons. Inhibition of ABHD6 has been shown to reduce excessive excitation during seizures by increasing 2-AG levels and allosterically increasing GABA_A_ receptor activity.^38,216–218^ Beyond the canonical mechanisms of 2-AG-mediated control of synaptic function, 2-AG is also the starting point of another lipid signaling pathway. MAGL-mediated hydrolysis of 2-AG yields the cyclooxygenase-2 substrate arachidonic acid,^219^ and this eCB source of substrate plays a dominant role in brain prostaglandin production.^220^ In light of this finding, MAGL inhibitors have been tested in mouse disease models and demonstrated to ward off neurodegeneration in a model of Parkinson’s disease^220^ and neuropathological features in a model of Alzheimer’s disease.^221^ This dual role for synaptic and neuroinflammatory control by 2-AG is perhaps best exemplified during seizures, where activity-dependent production of 2-AG is hijacked by excessive neural activity to produce supraphysiological 2-AG levels. On one hand, dramatic 2-AG elevations can attenuate seizures via the CB_1_ receptor, which is consistent with work performed in a range of mouse models.^222–225^ Conversely, elevated 2-AG fuels prostaglandin signaling pathways,^220^ which were found to drive profound vasoconstriction after seizures, resulting in over an hour of severe local brain hypoxia.^3^ This work exemplifies the Janus-faced nature of 2-AG in some disease settings, given the potential benefit of synaptic signaling in restricting hyperexcitability and potential detriment of prostaglandin-mediated control of cerebral blood flow. In general, more work is needed to better understand how the multiple roles of 2-AG contribute to disease mechanisms. Utilizing biosensors to visualize the spatiotemporal dynamics of 2-AG and its downstream metabolites in different cell types, including microglia and astrocytes,^216,226,227^ alongside molecular profiling to determine enzyme and gene expression changes in disease could elucidate underlying mechanisms and therapeutic strategies. Moreover, MAGL inhibitors could prove to be a useful tool in augmenting 2-AG levels when and where they are locally produced, potentially leading to diminished side effects associated with CB_1_ receptor agonism, but can also restrict prostaglandin production and suppress the neuroinflammatory components of neurological and psychiatric disease.^228^ Utilization of biosensors has the potential to expand our knowledge about exogenously applied cannabinoids (exocannabinoids) as well. For example, the eCB sensor offers the possibility to visualize the modulation of presynaptic terminals by the psychoactive component of marijuana, delta-9-tetrahydrocannabinol (THC), that acts as a weak partial agonist at CB_1_ receptors, in various key reward- and cognition-related brain areas during behavior. Similarly, these new tools open novel opportunities to refine our understanding of how CBD, the first marijuana-derived compound to be approved for medicinal use in children with devastating forms of epileptic encephalopathy,^229,230^ may exert its beneficial effects *in vivo*.^231^ Finally, these approaches could now be employed to investigate exciting novel therapeutics with intracellular signaling-specific mechanisms of action, such as the possibility of inhibition of THC effects without producing behavioral effects per se for cannabis use disorder.^41^

## CONCLUSION

As illustrated by the results highlighted in this review, tremendous progress has been made in gaining an integrative and translational understanding of the eCB system. The development of the GRAB_eCB_ sensor allowed for the *in vivo* imaging of eCBs at unprecedented spatiotemporal resolution and provided insights into their physiological roles. Identification of a specific marker (Sncg) for CCK- and CB_1_ receptor-expressing INs allowed for the first population-level study of these cells in awake, non-anesthetized animals, both in terms of activity monitoring and also by providing genetic access for their optogenetic and chemogenetic manipulations. The combination of the approaches represented by the eCB sensor and the Sncg transgenic mouse line then allowed for the first *in vivo* demonstration of DSI and showcased the importance of this phenomenon in place field formation. Finally, as the outstanding questions we have presented demonstrate, much remains to be discovered about the eCB system at the levels of molecules, cells, and behavior. There is an abundance of evidence pointing to the potential roles of the eCB system in various pathological states, but pinpointing therapeutic targets will require a multi-system approach that can begin to determine how disruptions of eCB molecular signaling pathways can lead to changes in CB1 receptor-expressing microcircuits with brain state-dependent behavioral consequences. For a glossary of the terms used in this review, see Box 4.

## Box 1. Key properties of the eCB synaptic signaling system

ECBs are lipid-derived molecules that are synthesized postsynaptically but act on presynaptically located CB_1_ receptors. The two major eCBs in the brain are 2-AG and AEA (see panel A in box image), with 2-AG being a full CB_1_ receptor agonist and AEA a partial agonist.^14^ The eCB system is implicated in various physiological functions, and CB_1_ receptors are the most abundant GPCRs in the brain.^6^ Importantly, eCB signaling is under tight spatiotemporal control by 2-AG and AEA synthesis and breakdown enzymes (B).^15–21^ Furthermore, the differential expression of CB_1_ receptors across brain regions impacts the behavioral effects of CB_1_ receptor ligands. For example, the high CB_1_ receptor expression within the ventral tegmental area, nucleus accumbens, and the prefrontal cortex is involved in reward-related processing (C).^2^ The levels and patterns of CB_1_ receptor expression across brain areas contribute to the characteristic effects of CB_1_ receptor agonist administration on whole-organismal physiology, collectively known as the “cannabinoid triad”: anti-nociception, hypothermia, and catalepsy (D).^22^

At a cellular level, CB_1_ receptors in cortical circuits are mainly expressed on CCKBCs, where they regulate GABA release via DSI, and to a lesser extent on pyramidal cells (PCs), where they regulate glutamate release via DSE. In the case of DSI, which is a focus of this review, a depolarization-dependent increase in intracellular calcium in the postsynaptic cell leads to the production and subsequent release of 2-AG, which then retrogradely (i.e., postsynaptic to presynaptic) activates presynaptic CB_1_ receptors to suppress GABA release^23–28^ (E and F). Notably, CB_1_ receptors often display the property of baseline or constitutive signaling that is independent of eCBs, referred to as tonic inhibition (G). This constitutive activity of the CB_1_ receptor is a powerful regulator of GABA release from CCKBCs and can even lead to “silent inhibitory synapses”^10,28–30^ (G).

## Box 2. Challenges using intersectional transgenic approaches for labeling CCK cells

A method that sometimes is used to attempt to target CCK INs involves double conditional labeling of CCK-expressing and Dlx-expressing cells (i.e., GABAergic INs).^63^ This is done using a double transgenic CCK-Cre/Dlx-Flp mouse line or through the injection of CCK-Cre mice with a Cre-dependent Dlx-promoter virus. Note, however, that such methods are sensitive to even very low levels of Cck gene expression to cause recombination and lead to positive cell labeling. This low detection threshold of conditional genetic approaches is distinct from the relatively high detection threshold of CCK octapeptide immunostaining, which was used in classical neuroanatomical studies to define CCK-expressing cells.^64^ The Cck gene is often expressed at low levels by PCs and several IN subtypes,^65^ and therefore methods relying on Cck for genetic targeting often result in labeling various cell types, including not only CCK INs but also PV INs. For example, a Cck/Dlx intersectional approach results in only 14% of the labeled cells being genuine CCK INs (with the latter being assessed by the more specific proCCK immunostaining), whereas 25% were PV INs.^66^ Another recent study using a similar approach to study CCK INs found 19% of labeled cells to be positive for PV.^67^ In the amygdala, 29% of labeled “CCK” neurons expressed neuropeptide Y, and 17% expressed PV.^68^ Other studies noted that Cck was not useful in targeting specific cell types without presenting the data,^69^ indicating that unsuccessful validation efforts can remain unpublished due to a bias against publishing negative results. Thus, results from studies that have used approaches based on the Cck-Cre strategy (including Dlx intersectional approaches) in the hopes of specifically targeting CCK cells in order to examine the dichotomy and distinct characteristics of PV and CCK INs should be interpreted with these caveats in mind.

## Box 3. Molecular and cellular dichotomy of PVBCs and CCKBCs

PVBCs and CCKBCs exhibit dichotomous properties that are thought to give them unique, non-overlapping roles in circuits (for review, see Armstrong and Soltesz^77^). PCs receive convergent input from two BC populations, PVBCs and CCKBCs. PVBCs are characterized by a fast-spiking firing pattern, while CCKBCs are adapting, regular-spiking (see panel A in box image). PVBCs receive strong feedforward (from CA3) and feedback excitation (A). By contrast, CCKBCs receive considerably fewer excitatory synapses in general, although indirect evidence suggests that they may be driven by subsets of fibers from CA2 and lateral entorhinal cortex (LEC). PVBC action potential firing is followed by precisely timed, so-called synchronous inhibitory postsynaptic currents (IPSCs) on the postsynaptic PC (A). By contrast, CCKBC firing evokes less precisely timed, asynchronous IPSCs that linger after presynaptic activity. Unique expression patterns of calcium-binding proteins^78^ may contribute to some of these differences in IPSC synchrony. The higher levels of excitatory inputs, fast-spiking pattern, and non-passive dendritic cable properties make PVBCs well-equipped to respond quickly to incoming input. On the other hand, the cell-intrinsic properties of CCKBCs, together with fewer excitatory inputs, make these cells more responsive to combined inputs. The functions of PVBCs and CCKBCs have often been generalized to being the “timekeepers” and “modulators,” respectively, of the network.^79^ As this review will continue to show, the function of CCKBCs goes far beyond this simple definition. Further highlighting their dichotomy, PV cells originate from the medial ganglionic eminence (MGE), whereas CCK cells arise from a distinct part of the embryonic brain called the caudal ganglionic eminence (CGE, B).

## Box 4. Glossary

**2-AG**: one of the two major eCB ligands in the brain and likely the primary ligand generated in response to hippocampal neural activity.

**ABHD6/ABHD12**: enzymes responsible for a small percentage of 2-AG breakdown.

**AEA**: one of the two major eCB ligands in the brain. The functions and dynamics of this ligand are less known compared with 2-AG.

**AOD 3D imaging**: an imaging technique that allows for imaging of large volumes of brain tissue at a high resolution. It is often used to capture activity fluctuations of a greater number of cells than other imaging modalities allow. For more details, see Katona et al.^81^ and Geiller et al.^70^

**BARR**: high frequency and persistent firing of action potentials.

**BTSP**: a form of synaptic plasticity that occurs on behaviorally relevant, seconds-long timescales. Specifically, BTSP is induced by dendritic plateau potentials, which are large calcium events occurring in dendrites, and enables *de novo* place field formation.

**CB**_**1**_ **receptor**: the primary cannabinoid receptor in the central nervous system. Its counterpart, the CB2 receptor, is primarily found in the peripheral nervous system.

**CBD**: a non-psychoactive compound found in cannabis.

**CCK**: a peptide found throughout the brain and body with modulatory and neuromodulatory properties.

**CCKBC**: a type of perisomatically targeting GABAergic IN that expresses high levels of CCK. CCKBCs are also uniquely known to be the primary IN expressing CB1 receptors.

**CGE**: a transient structure present in the developing brain important for cell migration and production of GABAergic INs. CCK INs are derived from the CGE.

**DAGLα/DAGLβ**: 2-AG synthesis enzymes.

**DSA INs**: INs in the cortex that are most active during NREM sleep. Notably, their activity is anticorrelated with PCs and other INs.

**DSE**: depolarization of a postsynaptic cell leads to reduced glutamate release from a nearby presynaptic axon terminal. In the case of the eCB system, depolarization of a PC leads to synthesis of eCBs, which act on excitatory presynaptic CB1 receptor-expressing terminals. This leads to reduced glutamate release from the presynaptic terminals.

**DSI**: depolarization of a postsynaptic cell leads to reduced GABA release from a nearby presynaptic axon terminal. In the case of the eCB system, depolarization of a PC leads to synthesis of eCBs, which act on inhibitory presynaptic CB1 receptor-expressing terminals. This leads to reduced GABA release from the presynaptic terminals.

**eCBs**: referring to endogenous molecules (2-AG and AEA) that primarily act on cannabinoid receptors (CB_1_ and CB_2_ receptors).

**GABAergic IN**: locally projecting neuron that releases GABA.

**GDP**: spontaneous, synchronous GABAergic depolarizing events that occur during development.

**GPCR**: a cell surface receptor that, upon ligand binding, interacts with a G-protein in the plasma membrane. The G-protein then interacts with a variety of secondary messengers (depending on the type of GPCR) to modulate cellular function.

**iLTD**: a form of synaptic plasticity characterized by reduced synaptic strength that is induced *in vitro*.

**IPSC**: flow of ions across the cell membrane representing an inhibitory event.

**IPSP**: a small hyperpolarizing change in voltage across the cell membrane.

**LEC**: responsible for providing multisensory input to the hippocampus, likely playing a role in nonspatial contextual coding of memories. The medial entorhinal cortex, on the other hand, provides more spatial coding information.

**MAGL**: primary enzyme responsible for 2-AG breakdown.

**MGE**: like the CGE, the MGE is a transient structure present in the developing brain important for cell migration and production of GABAergic INs. PV INs are derived from the MGE.

**NREM**: a period of the sleep cycle consisting of three stages (N1, N2, and N3) that encompass the transitions from the beginning of sleep to deep sleep.

**PC**: named for the pyramidal shape of its soma, this is a type of excitatory neuron. Notably, it is the primary excitatory neuron in the CA1 of the hippocampus.

**PV**: an intracellular protein with calcium-binding sites. It is primarily expressed in a specific subset of INs.

**PVBC**: a type of perisomatically targeting GABAergic IN that expresses high levels of PV.

**Sncg/Sncg-Flp**: a gene that is selectively expressed by VGLUT3 CCKBCs. Discovery of this gene’s selectivity for CCKBCs led to the development of the Sncg-Flp mouse line.

**SPW-Rs**: sharp waves are large amplitude events in the stratum radiatum that are often associated with quick bursts of oscillatory activity, known as ripples, in the stratum pyramidale. Together, these events are known as SPW-Rs.

**THC**: the primary psychoactive compound in cannabis.

**TORO cells**: hippocampal long-range projecting GABAergic cells that are ripple selective, locally innervate PV and CCK INs, and project to extra-hippocampal regions. For more detail, see Szabo et al.^82^

**V1/V2**: subregions within the occipital lobe responsible for visual information processing.
